# Electronic screening through community engagement: A national strategic plan to find COVID-19 patients and reduce clinical intervention delays

**DOI:** 10.1017/ice.2020.188

**Published:** 2020-05-04

**Authors:** Mehrdad Amir-Behghadami, Masoumeh Gholizadeh

**Affiliations:** 1 Tabriz Health Services Management Research Center, Health Management and Safety Promotion Research Institute, Tabriz University of Medical Sciences, Tabriz, Iran; 2 Iranian Center of Excellence in Health Management (ICEHM), Department of Health Service Management, School of Management and Medical Informatics, Tabriz University of Medical Sciences, Tabriz, Iran; 3 Student Research Committee (SRC), Tabriz University of Medical Sciences, Tabriz, Iran

*To the Editor—*Coronavirus disease 2019 (COVID-19) began circulating in Wuhan, Hubei Province, China, in December 2019. Evidence of human-to-human transmission has been reported in both communities and hospitals^1^; COVID-19 is a highly contagious disease that can spread rapidly through respiratory droplets of infected individuals.^2^ According to one of the first published COVID-19 studies, the most common symptoms at onset are fever, cough, myalgia, and/or fatigue, and less common symptoms include sputum, headache, hemoptysis, and diarrhea.^3^ However, these symptoms may be more severe in the elderly, the immunosuppressed, and those with chronic diseases including diabetes, cardiovascular diseases, cancer, and pulmonary dysfunction.^4,5^

The global pandemic is evolving dynamically. On January 30, 2020, the World Health Organization (WHO) declared that COVID-19 is a “Public Health Emergency of International Concern (PHEIC)” during its second meeting of the Emergency Committee.^6^ As of February 27, 2020, there were 1,610,909 accumulative confirmed cases and 99,690 cumulative deaths globally.^7^ Iranian health authorities confirmed the first COVID-19 cases on February 19, 2020, in Qom. As of February 27, 2020, there had been >66,220 confirmed cases of COVID-19 and 4,110 deaths in Iran.^8^ Notably, however, Iran has had the highest improvement in COVID-19; it ranks second in the world after China. Recently, although the number of new cases reported in China has been steadily decreasing, epidemics in other countries are still a major concern. Prevention and identification of the disease have become the most important tasks in Iran, and the government has invested many material and human resources to manage the epidemic.^9^ Currently, no licensed preventative vaccine or specific antiviral therapy is available for COVID-19, and according to the basic theory of controlling infectious diseases, the most effective measures include eliminating the source of infection, disrupting transmission, and protecting susceptible individuals. Therefore, to cope with a sudden outbreak of COVID-19, the community needs to be screened, and whether the infection has occurred and the dynamics of when it is contagious need to be understood more fully.

The Iranian Ministry of Health and Medical Education designed and has been implementing an electronic national screening system (https://salamat.gov.ir/) using a modern information network technology.^10^ After logging information (eg, national code, date of birth, phone number) into the system, Iranian residents answer some questions about COVID-19 symptoms, immunosuppression, and some chronic diseases, as well as the presence of others suspected of having COVID-19 disease among their relatives. Those suspected of having the disease receive a message regarding their health status, and healthcare providers then call them and guide them. Also, the their residences are disinfected and other family members are quarantined if required. If they do not improve within 3 days, they are referred to the emergency department of a hospital. Some screening-related information is provided in Table 1.

**Table 1.** Indicators of Screened, Infected, and Death Cases

Screening (March 4 through April 7, 2020)	Value
No. of services delivered for COVID-19	70,141,824
Target population with symptoms, %	1.45
Screened symptomatic cases referred to clinic, %	0.2
Visited cases that required homecare, %	23.9
Visited cases that received dual medication, %	0.6
Visited cases that referred to the hospital, %	4.5
People visiting hospital and are admitted in the hospital, %	29.2
Client satisfaction with the received services, %	97.3
**New lab-confirmed cases (in the previous 72 h)**	
Age, mean y (standard deviation)	54.8 (18.5)
Age, median y (interquartile range)	55 (39–68)
**Sex distribution, %**	
Male	49.6
Female	50.4
Cases with at least 1 comorbidity, %	26.1
Cases admitted in ICU, %^a^	11.6
Cases with more severe forms of the disease, %^b^	15.4
**COVID-19 deaths (in the previous week)** ^c^	
Age, mean y (standard deviation)	69.6 (15.1)
Age, median y (interquartile range)	71 (61–81)
Cases aged >60 y, %	77.3
**Sex distribution (%)**	
Male	58.4
Female	41.6
Cases with at least 1 comorbidity, %	44.2
Cases aged >60 y with at least 1 comorbidity, %	87.7

Note. COVID-19, coronavirus 2019; ICU, intensive care unit.

a To the total number of hospitalized COVID-19 patients.

b Based on available data, we considered patients with death outcome, as well as those admitted to ICU or under mechanical ventilation, as more severe cases. The information in this chart is based on hospitalized cases, and outpatients are not included in this calculation. Inclusion of outpatients and asymptomatic cases would decrease the proportion of severe cases.

c To increase the sample size, the analysis of death cases was performed using the data from the previous week. Reference: ***Daily Situation Report on COVID-19, Ministry of Health and Medical Education, IR Iran.***

This self-screening plan has been successful through government implementation and community engagement. During the pandemic, many efforts have been made to find effective and efficient solutions for the initial management of COVID-19 globally. One of the significant factors, which is been emphasized today, is the important role of community engagement in the management and screening of infected patients. People's attitudes toward the disease and understanding of its consequences if left untreated have played an important role in encouraging their participation in self-screening through designed website. The government has allocated >17,000 health houses and >9,000 comprehensive health centers in urban, suburban, and rural areas throughout the country to support the plan. These centers, as community health centers, play an important role in these efforts; they are responsible for delivering integrated care services to the population in geographically defined areas.^11^ In addition, this plan is consistent with the overall goal of developing health systems, strengthening their capacity to meet the needs of the community, and achieving universal health coverage.

In conclusion, a successful electronic screening system was developed and introduced to combat the COVID-19 pandemic in Iran. On one hand, this system helps in the initial identification of patients with COVID-19 infections and prevents any delay in clinical interventions. On the other hand, it prevents nonemergency referrals to emergency departments of hospitals. Implementing a simple strategy can be effective for the health system in dealing with this pandemic. Therefore, sharing our successful experience, which was the result of good cooperation and cohesion between the government and community, may be helpful for authorities in other countries.
